# Breaking barriers: unraveling the impact of cultural beliefs and misconceptions on HPV vaccination uptake—the narrative review

**DOI:** 10.3389/fpubh.2026.1788012

**Published:** 2026-03-25

**Authors:** Maryam Abdulla, Syeda Mehreen Shah, Wajd Abosoudah, Seham Bader, Syeda Maham Fatima Shamsi, Rahaf Aldiab, Batool Alsafadi, Sarah Ahmed Yaqinuddin, Suha Syed Mohsin Hussain, Laraib Mohammad Faisal Chishtee, Baraa Alghalyini, Abdul Rehman Zia Zaidi

**Affiliations:** 1 College of Medicine, Alfaisal University, Riyadh, Saudi Arabia; 2 Department of Family and Community Medicine, College of Medicine, Alfaisal University, Riyadh, Saudi Arabia

**Keywords:** cultural barriers, global health, HPV vaccination, human papillomavirus, misconceptions, prevention, public health policy, vaccine hesitancy

## Abstract

**Background:**

Human papillomavirus (HPV) vaccination represents a critical public health intervention for preventing HPV-related cancers, yet global uptake remains suboptimal due to cultural beliefs and misconceptions. This narrative review examines the multifaceted barriers to HPV vaccination acceptance across diverse populations.

**Methods:**

We conducted a comprehensive literature search of PubMed, Scopus, Web of Science, and Google Scholar for articles published between January 2010 and December 2025. Search terms included combinations of HPV vaccination, cultural beliefs, misconceptions, barriers, and vaccine hesitancy. Articles were screened for relevance to cultural and social determinants of HPV vaccine uptake.

**Results:**

Cultural and religious beliefs significantly influence HPV vaccination decisions across global populations. Common misconceptions include concerns about vaccine safety, fears regarding sexual promiscuity, and misinformation about vaccine efficacy. Structural barriers such as healthcare access, cost, and provider recommendation practices compound these challenges. Successful intervention strategies incorporate community engagement, culturally tailored education, and healthcare provider training.

**Conclusion:**

Addressing HPV vaccination barriers requires multifaceted approaches that respect cultural contexts while providing evidence-based education. Future research should focus on developing and evaluating culturally sensitive interventions across diverse populations.

## Introduction

### Background on HPV and vaccination

Human papilloma virus (HPV) is a sexually transmitted pathogen etiologically linked to multiple cancers and anogenital warts. Vaccination remains the most effective primary prevention strategy against HPV-associated diseases (1). Nevertheless, persistent misconceptions regarding vaccine safety and its role in cancer prevention continue to discourage uptake across diverse populations (2). This paper explores these factors alongside the broader cultural influences that impede HPV vaccine adoption. Understanding how cultural barriers shape vaccination behavior is essential for tailoring public health programs toward achieving universal and equitable immunization coverage.

HPV encompasses over 200 genotypes, several of which are oncogenic and linked to cervical, oropharyngeal, anal, and penile cancers (2). In the absence of vaccination, the vast majority of sexually active individuals will contract at least one HPV genotype during their lifetime. HPV-related conditions are particularly prevalent among those aged 15–25 years in the United States (3). Current clinical guidelines recommend initiating HPV vaccination for both males and females at ages 11 or 12 years (4), although schedule may be adjusted according to age, medical history, vaccine formulation, and sex (5).

### Prevalence of HPV and the purpose of the review

**Figure 1:**
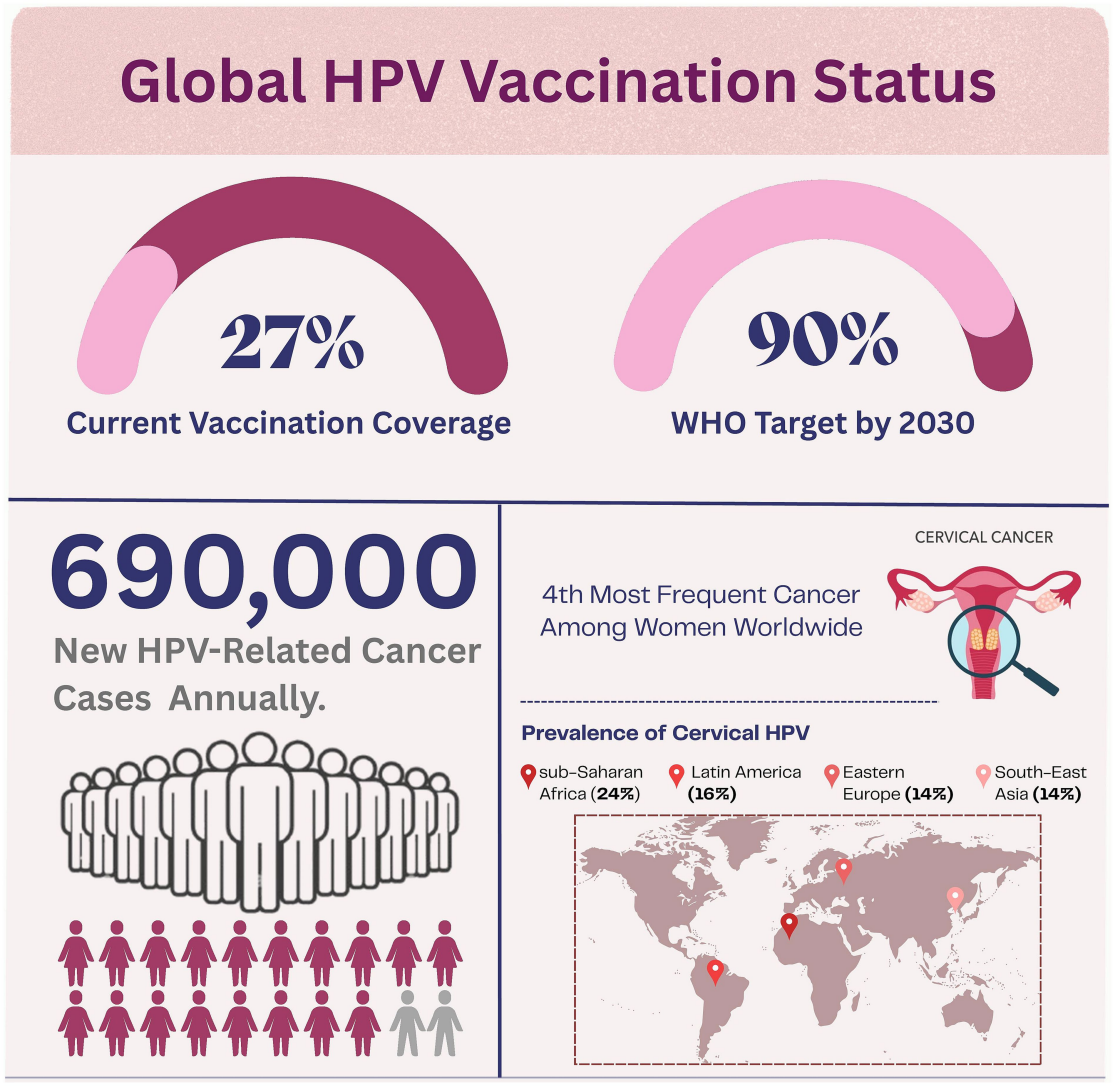
Global HPV vaccination status, cervical cancer burden, and regional HPV prevalence. This figure illustrates the global HPV vaccination status, showing that 27% of girls received the first dose of the HPV vaccine, far short of the WHO’s target of 90% coverage by 2030. It quantifies the annual impact of 690,000 new HPV-related cancer cases. The figure also highlights that cervical cancer, mostly caused by chronic HPV infection, is the fourth most frequent cancer among women worldwide. The prevalence of cervical HPV is highest in sub-Saharan Africa (24%), followed by Latin America and the Caribbean (16%), Eastern Europe (14%), and South-East Asia (14%).

Globally, HPV constitutes a substantial public health burden. In 2022, an estimated 660,000 new cases of cervical cancer and 350,000 associated deaths were reported worldwide, rendering cervical cancer the fourth most common malignancy among women (3). Collectively, HPV-attributable cancers across both sexes account for approximately 690,000 new cases annually (3). The prevalence of cervical HPV is highest in sub-Saharan Africa (24%), followed by Latin America and the Caribbean (16%), Eastern Europe (14%), and South-East Asia (14%) (3). Despite the effectiveness of prophylactic vaccination against HPV in preventing these cancers, global vaccination coverage is still low. In 2023, only 27% of girls received the first dose of the HPV vaccine, far short of the WHO’s target of 90% coverage by 2030 (3). Figure 1 illustrates the current global HPV vaccination landscape, highlighting the significant gap between current coverage (27%) and WHO targets (90% by 2030). The figure also demonstrates the substantial annual burden of 690,000 new HPV-related cancer cases and shows regional variations in cervical HPV prevalence, with sub-Saharan Africa having the highest rates at 24%.

There is a rapid increase in HPV-associated cancers due to the low rates of vaccination in America (6). Therefore, it is crucial to find out if cultural beliefs and misconceptions contribute to vaccination hesitancy (7). The fragmented vaccination usage has perpetuated several disparities across diverse American communities (6, 8). Augmenting vaccination efficacy makes sure that all communities, individuals, and children live happier, healthier lives (1). This paper aims to explore cultural indices and immunization perceptions within various communities, examine the barriers created by such factors, identify sustainable solutions to these problems, and compare the effectiveness of the solutions across different communities. This review will enhance vaccination uptake since it will facilitate more culturally attentive and sensitive interventions.

### Scope and structure

Promoting early prevention and detection of HPV and its risks is a call for optimal public health. Vaccination represents an imperative step toward reaching this goal (1). Cultural perceptions and beliefs can impact vaccination confidence. Some communities value modesty, thus making discussions about sexual health awkward and uncomfortable, which can hinder HPV vaccination uptake (9). Vaccine policies are developed by acknowledging and accommodating cultural differences to encourage more people to receive the vaccines. Traditional viewpoints on medical treatment can also dampen vaccination uptake (1). In a collaboration between informed healthcare providers and parents regarding HPV vaccination, there was consequently a positive response to vaccine uptake, thus demonstrating that there is a need to raise positive beliefs and awareness in the entire community powered by healthcare workers (10).

Social media exerts considerable influence on public perception of HPV vaccination. Parental beliefs that adolescents do not require vaccination adversely affect uptake (11), while anti-vaccination sentiments, including unfounded claims that HPV vaccines cause infertility or autism, have gained traction in public discourse (12, 13). Despite the absence of supporting evidence, such misconceptions persist, fueling vaccine opposition and diminishing adoption rates (14). Governmental transparency and accountability in disseminating vaccine-related knowledge may help mitigate these effects (15).

This narrative review aims to: (1) identify and critically examine the cultural beliefs, misconceptions, and sociocultural factors that contribute to suboptimal HPV vaccination uptake across diverse global settings; (2) assess the impact of these cultural determinants on vaccination behavior, with attention to regional and demographic variations; and (3) evaluate evidence-based strategies and interventions that have demonstrated effectiveness in overcoming cultural barriers to HPV vaccination, with the goal of informing culturally sensitive public health policy and practice.

## Methods

This study was conducted as a narrative review, guided by the Scale for the Assessment of Narrative Review Articles (SANRA) framework (16). A comprehensive literature search was performed across PubMed/MEDLINE, Scopus, Google Scholar, and Web of Science for articles published between January 2010 and December 2025. The search strategy employed combinations of the following terms: ‘human papillomavirus,’ ‘HPV vaccination,’ ‘vaccine hesitancy,’ ‘cultural barriers,’ ‘religious beliefs,’ ‘misconceptions,’ ‘vaccine uptake,’ and ‘health literacy.’ Boolean operators (AND, OR) were used to combine search terms during the search. Reference lists of identified articles were manually screened to capture additional relevant studies. Studies were included if they addressed sociocultural, religious, or psychosocial determinants of HPV vaccination uptake in any geographic setting and were published in English in peer-reviewed journals. Governmental and organizational reports from the WHO and CDC were also included where relevant. Editorials, conference abstracts, and commentaries were excluded. Two reviewers (ARZ, MA) independently screened titles and abstracts for relevance, with disagreements resolved through discussion and consensus. Data were synthesized thematically according to the major barriers and facilitators identified across studies. Given the narrative nature of this review, a formal quality assessment of individual studies using standardized tools (e.g., Newcastle-Ottawa Scale or JBI checklists) was not performed. However, preference was given to peer-reviewed studies published in indexed journals with clearly described methodologies. The findings are synthesized thematically rather than quantitatively, organized around the following domains: (a) global and regional vaccination trends, (b) cultural beliefs and vaccination perception, (c) misconceptions about the HPV vaccine, (d) barriers to HPV vaccination, (e) strategies to overcome cultural barriers, and (f) regional case studies and comparative analysis.

A total of 59 peer-reviewed studies met our inclusion criteria and were included in this narrative review. These studies encompassed cross-sectional surveys, systematic reviews, qualitative investigations, randomized controlled trials, and program evaluations conducted across diverse geographic regions. Table 1 presents a summary of the characteristics of all included studies, organized by geographic region, including study design, population, sample size, and key findings related to cultural barriers to HPV vaccination.

**Table 1:** Characteristics of studies included in this narrative review on cultural barriers to HPV vaccination.

#	Author(s), year	Country/region	Study design	Population	Sample	Key findings related to cultural barriers
A. Saudi Arabia and gulf cooperation council studies (*n* = 12)						
1	Alghalyini et al., 2024	Saudi Arabia	Cross-sectional survey	College students	*N* = 442	54.1% aware of HPV; only 10% vaccinated; 80.1% cited lack of education as primary barrier
2	AlShamlan et al., 2024	Saudi Arabia	Cross-sectional survey	Female healthcare workers	*N* = 1,857	20% received HPV vaccine; 45% willing to vaccinate; lack of knowledge top hesitancy reason
3	Aldawood et al., 2023	Saudi Arabia	Cross-sectional survey	Health college students	*N* = 405	49.9% aware of HPV vaccine; 5.2% vaccinated; 75.9% hesitancy in males vs. 43.9% in females
4	Hussain et al., 2016	Saudi Arabia	Cross-sectional survey	Female patients aged 11–26	*N* = 325	34.5% aware of HPV; Saudi nationals less aware (29.9%) than non-Saudis (48.8%)
5	Tobaiqy et al., 2023	Saudi Arabia	Cross-sectional study	Parents at university hospital	*N* = 500	96.8% never heard of HPV vaccine; 94% unwilling to vaccinate; 85.2% cited lack of information
6	Sulaiman et al., 2023	Saudi Arabia	Cross-sectional survey	Patients at King Saud Medical City	*N* = 384	Significant link between education level and HPV knowledge; lower education = less awareness
7	Almatrafi et al., 2024	Saudi Arabia	Interventional study	Secondary school girls	*N* = 148	Post-education knowledge rose from 43.9 to 94.6% (p < 0.001); cultural stigma identified
8	Moshi et al., 2024	Saudi Arabia	Mapping review	General population	Review	National uptake only 7.6%; 84.1% lacked cervical cancer screening knowledge
9	Mahmoud et al., 2024	GCC countries	Comparative cross-sectional	Young adults aged 18–39	*N* = 831	UAE highest vaccination (18.9%), KSA only 4.6%; 53.6% cited lack of knowledge
10	Zakhour et al., 2023	Lebanon	Cross-sectional KAP survey	Parents of children	*N* = 306	60% would not vaccinate; lack of physician recommendation top reason; gender bias present
11	Elbarazi et al., 2016	United Arab Emirates	Content analysis	Newspaper coverage	Media 2006–2014	Media coverage limited; taboo topics like promiscuity not addressed in coverage
12	Kisa and Kisa, 2024	OIC countries	Scoping review	Multiple populations	23 studies	Religious misconceptions drove hesitancy; 30% of Saudi opposition religiously motivated
B. Sub-Saharan Africa studies (*n* = 4)						
13	Kutz et al., 2023	Sub-Saharan Africa	Systematic review	Communities, parents, adolescents	20 studies	Barriers: limited health systems, stigma, cost, misinformation; HPV prevalence 24%
14	Turiho et al., 2017	Uganda	Qualitative study	Schoolgirls, parents, teachers	*N* = 105	Rumors: vaccine causes infertility, is population control; religious group opposition
15	Binagwaho et al., 2012	Rwanda	Program evaluation	Primary school girls	*N* = 93,888	Achieved 93.23% three-dose coverage; school-based delivery with community outreach
16	Cooper et al., 2024	Africa (multiple)	Research synthesis	Communities	Multiple studies	Social media can build vaccine confidence; community engagement and social science approaches effective
C. Asia-Pacific studies (*n* = 7)						
17	Taghizadeh Asl et al., 2020	Iran	Qualitative study	Married women aged 25–65	*N* = 81	Near-total HPV ignorance; cultural stigma around sexual organs; health deprioritization
18	Tay et al., 2015	Singapore	Cross-sectional survey	Female nurses	*N* = 1,611	38.9% believed vaccine experimental; 76% believed cultural practices influenced health decisions
19	Wong et al., 2019	China	Narrative review	General population	Review	Barriers: high cost, limited supply, preference for 9-valent vaccine; need school-based programs
20	Krokidi et al., 2023	India	Literature review	People aged 9–29	7 studies	Health education effective; barriers: cost, awareness, STI stigma, patriarchal norms
21	Mehra et al., 2025	India	Infodemiological study	Google Trends 2010–2024	Search data	Significant increase in HPV vaccine searches after policy changes; regional variations
22	Njogu et al., 2024	Kenya, India, Nigeria	Multi-country qualitative	Healthcare workers	Multiple sites	Effective HCW communication strategies; need culturally tailored messages
23	Islam et al., 2024	India	Quantitative analysis	Children and families	Population data	Patriarchal norms negatively impact child wellbeing; gender-based barriers to healthcare
D. United States studies (*n* = 14)						
24	Hirth J, 2019	United States	Literature review	US population	Review	Significant disparities by race/ethnicity, geography, socioeconomic status
25	Rahman et al., 2015	United States	Cross-sectional analysis	Young adults aged 18–26	*N* = 3,727	Southern women lowest initiation (30.4%) vs. Northeast (58.7%); male vaccination 6.3%
26	Xiong et al., 2024	United States	Cross-sectional analysis	Male and female children	5 states	Geographic-based socioeconomic factors significantly associated with HPV vaccination
27	DiClemente et al., 2015	United States	Randomized clinical trial	African American females	*N* = 216	Culturally-tailored media intervention improved compliance; perceived susceptibility key
28	Adegboyega et al., 2023	United States	Cross-sectional survey	African Americans, African immigrants	*N* = 200	Knowledge gaps identified; African immigrants had lower awareness; cultural beliefs influential
29	Harrington et al., 2021	United States	Narrative review	Racial/ethnic minorities	20 articles	High trust in doctors but low in pharma; mistrust associated with lower uptake
30	Morales-Campos et al., 2021	United States	Qualitative study	Mexican American adults	8 focus groups	Gendered perspectives: fathers linked risk to female promiscuity; cultural taboos present
31	Gilkey et al., 2016a	United States	Cross-sectional survey	Parents of adolescents	*N* = 1,495	High-quality recommendations: 9-fold odds of initiation; only 36% received quality recommendation
32	Gilkey and McRee, 2016	United States	Systematic review	Healthcare providers, patients	101 studies	Providers less likely to recommend if uncomfortable discussing sex or perceived hesitancy
33	Gilkey et al., 2015	United States	National survey	Physicians	*N* = 776	Quality of physician communication varied; strong endorsement associated with higher uptake
34	Oh et al., 2021	United States	Meta-analysis	Parents and adolescents	Multiple studies	Provider recommendation strongly associated with uptake; quality and timing matter
35	Sundstrom et al., 2021	United States	Campaign evaluation	General public	Campaign data	HPV Vaccination NOW campaign effective at correcting misinformation online
36	Bruns et al., 2024	United States	Cross-sectional survey	University community	*N* = 1,539	Knowledge and trust significantly associated with vaccine confidence; education gaps identified
37	Brandt et al., 2016	United States	Policy analysis	Policy makers	Policy review	Policy interventions effective; school-entry requirements and insurance mandates increase uptake
E. global and multi-country Studies (*n* = 14)						
38	Bruni et al., 2016	Global (64 countries)	Pooled analysis	Females in HPV programs	47 million	Global coverage 1.4%; 33.6% in developed vs. 2.7% in less developed regions
39	Spayne and Hesketh, 2021	Global (195 countries)	Cross-sectional analysis	Vaccine-eligible girls	61 million	Global coverage 12.2%; fewer than half of countries reported data; LMICs face barriers
40	Hopkins and Wood, 2013	Global	Cross-sectional analysis	Females targeted	Multiple countries	Vaccination lower in Asian/European countries; cultural attitudes impact uptake
41	Netfa et al., 2020	Multi-country (Western)	Systematic review	Immigrant parents	19 studies	11/16 studies found no HPV vaccine knowledge; religious abstinence belief key barrier
42	Graci et al., 2024	Global	Systematic review	Migrants and refugees	34 studies	Adherence 34.5% among migrants, 0.6% refugees; 58.8% cited health literacy barrier
43	Grandahl and Neveus, 2021	Global	Narrative review	Boys and young men	103 articles	Five barriers: lack of knowledge, hesitancy, absent recommendations, cost, promiscuity myth
44	Ortiz et al., 2019	Global	Systematic review	General population	44 articles	Social media improved awareness but not uptake; negative content associated with lower rates
45	Escoffery et al., 2023	Global	Systematic review	Adolescents, parents, HCPs	79 articles	Most interventions informational; initiation ranged 5–99.2%; only 33.8% used theory
46	Jarrett et al., 2015	Global	Systematic review	General population	Multiple studies	Dialogue-based interventions, reminders, education effective; multi-component approaches best
47	Kyei et al., 2024	Global	Conceptual analysis	HPV hesitancy studies	29 articles	False cultural beliefs primary antecedent (14/29 studies); perceived promiscuity key
48	Xu et al., 2024	Low-resource settings	Narrative review	Adolescents in LMICs	Review	Sociocultural barriers: promiscuity concerns, religious beliefs, gender norms
49	Enria et al., 2024	Global	Research synthesis	General population	Multiple studies	Political dimensions of misinformation; trust and vaccine confidence interlinked in digital age
50	Yim et al., 2024	Global	Narrative review	Immunization programs	Review	Sustainable financing challenges for immunization programs; funding models vary globally
51	Brewer et al., 2017	Global	Scientific review	Vaccination programs	Review	Presumptive announcements more effective than participatory approaches for vaccination
F. Vaccine science and general studies (*n* = 8)						
52	Williamson AL, 2023	Global	Scientific review	HPV vaccine development	Review	Recent developments in HPV vaccinology; different formulations may increase hesitancy
53	Clift and Rizzolo, 2014	United States	Review article	General population	Review	Main factors: safety concerns, religious objections, science skepticism; autism myth persists
54	Bezbaruah et al., 2024	Global	Book chapter	General population	Review	Common myths: vaccines cause disease, natural immunity superior, contain toxic materials
55	Hofstetter and Rosenthal, 2014	United States	Review article	Adolescents and parents	Review	Healthcare professional communication critical for STI vaccine acceptance including HPV
56	Rathod et al., 2023	Global	Comprehensive review	Women’s health	Review	HPV vaccination critical for cervical cancer prevention; need comprehensive strategies
57	Nielsen-Bohlman et al., 2004	United States	IOM Report	General population	Report	Health literacy impacts health outcomes; cultural values influence treatment concordance
58	Vehmas E, 2021	Global	Thesis/Review	General population	Review	Vaccination beliefs shaped by cultural aspects; need for culturally sensitive approaches
59	Salleh et al., 2025	Global	Qualitative systematic review	Parents of daughters under 18	Multiple qualitative studies	Cultural norms and values predominantly shaped parental vaccination decisions; fathers as decision-makers in patriarchal families created barriers; religious and ethnic factors influenced uptake

FGDs, focus group discussions; GCC, Gulf Cooperation Council; HCPs, healthcare providers; HCWs, healthcare workers; HPV, human papillomavirus; IOM, Institute of Medicine; KAP, knowledge, attitudes, and practices; LMICs, low- and middle-income countries; OIC, Organization of Islamic Cooperation; SSA, sub-Saharan Africa; STI, sexually transmitted infection. This table includes all peer-reviewed studies cited in this narrative review. Studies are organized by geographic region. Total included: 59 studies comprising cross-sectional surveys (*n* = 20), systematic/literature reviews (*n* = 19), qualitative studies (*n* = 5), narrative reviews (*n* = 8), program evaluations (*n* = 1), randomized controlled trials (*n* = 1), policy analyses (*n* = 2), and other study designs (*n* = 3). Government and organizational websites (CDC, WHO, etc.) are not included in this table.

## Results and discussion

### HPV vaccination: global and regional trends

#### Vaccination uptake and rates

Reports show that approximately 1 in 4 individuals are infected with HPV globally, with around 80 million Americans affected by the virus. In addition, over 36,500 Americans are diagnosed with an HPV-associated cancer every year (16). Also, there is evidence that HPV vaccination can prevent more than 90% of cancers caused by HPV (17). Despite the vaccine’s demonstrated ability to reduce persistent cervical HPV infections, precancerous lesions, and invasive cervical cancers (18), global efforts to integrate HPV vaccination into broader immunization programs and to encourage uptake across different age groups remain ongoing (19).

According to Kutz et al. (20), the prevalence of HPV-associated conditions in Africa, especially among women, is at 80% with respect to invasive cancers. Despite the HPV vaccine being tailored to address this problem, many countries have not yet incorporated it in routine immunization. Countries that have incorporated the vaccine view the HPV vaccine as an expensive undertaking. The World Health Organization states that 30–50% of cancers could be prevented if interventions were instituted (3). Moreover, the availability of HPV vaccinations in the continent is limited and culturally stigmatized, thus limiting its incorporation into healthcare systems for women (20).

According to a study by Bruni et al. (21), the global HPV vaccination coverage for developed countries was 56 percent, while that for developing countries was 22 percent. Most governments of developing nations are struggling to sufficiently vaccinate their populations. However, a later study showed that only 19.6 percent of the girls eligible for HPV vaccination had received the third dose of the HPV vaccine globally (22).

There are disparities in the rates at which vaccinations are offered or accepted in regions across the globe. Only over 25% of girls eligible for HPV vaccinations are administered with the vaccine in the U.S, with rates lower than this in many European and Asian countries (18). There are numerous HPV vaccination formulations available: Gardasil 9, Gardasil, and Cervarix (23). Different vaccine types have been shown to increase hesitancy among people as it leads to the doubt of which option is safer (24).

#### Factors influencing vaccination uptake

Numerous societal factors influence HPV vaccine uptake on a global scale. The limited availability of healthcare resources makes it crucial to identify which subpopulations are under-vaccinated and to explore the underlying causes of ethnic healthcare disparities (25). Researchers have acknowledged that healthcare professionals have been poorly informed about HPV vaccination and its impact (26). As a result, many women in developing areas present with advanced cervical cancers that go on to increase the mortality rate due to delayed diagnosis. It is important to include HPV in routine immunizations to cover this population. It is important for physicians to educate their patients about vaccinations offered and the need for adolescents to get vaccinated early free of autonomic symptoms. Education campaigns that are tailored to clarify misperceptions should be implemented, especially in developing nations (26).

Public education regarding the safety profile of HPV vaccines is fundamental to improving uptake (26). Enhanced knowledge among patients and caregivers may help address the persistently low vaccination rates observed globally. Although the role of mass media in shaping parental attitudes toward HPV immunization warrants further qualitative investigation, it is important to note that vaccine hesitancy is not solely attributable to knowledge deficits. Accordingly, vaccination campaigns emphasizing the public health importance of immunization may help improve coverage in middle- and high-income countries (27).

National policies are central in implementing the optimal healthcare services and HPV vaccination in schools. School immunization programs have improved vaccination rates in many parts of the U.S and worldwide (26). Routine secondary school attendance can play an important role in the knowledge levels and behaviors of mothers to immunize their daughters. The increased availability and participation of girls in school facilitates their access to healthcare services, amenities, and professionals who can clarify the necessity for immunization (18). Countries should adopt these policies to ensure all subpopulations understand the need for immunization against HPV (26). These policies should be tailored to address the healthcare disparities witnessed across many African countries and other developing nations.

Health equity demands that all population subgroups receive adequate education regarding HPV vaccination, irrespective of socioeconomic standing. Many low- and middle-income countries are constrained by insufficient healthcare infrastructure, a shortage of trained healthcare providers (26), and inadequate financing for immunization program implementation (20). Due to these problems, their healthcare costs have increased in the last two decades. Developing countries should seek assistance from large organizations and highly developed states, leading to more resource allocation to promote the HPV vaccination and cater to cervical cancer treatment outcomes (28).

### Cultural beliefs and vaccination perception

#### Understanding cultural beliefs

Cultural belief in sociology is defined as the shared values, standards, traditions, and behaviors that determine a specific society or group. Cultural beliefs are influenced by the cultural environment, which includes social, political, historical, and economic factors, which can influence individual and societal attitudes (29). It also includes factors like family dynamics and religious beliefs. According to Nielsen-Bohlman et al. (30), people’s wellbeing, health problems, health care system, and concordance with treatment plans and routine changes and recommendations are all influenced by cultural values and beliefs as well as the cultural environment. For example, some cultures prefer holistic approaches to health care where they believe there is a correlation between mental and physical health, so they prefer to follow conventional healing practices such as using herbal remedies, and meditation. Whereas other cultures ignore the individual mental health and wellbeing and focus on treating the clinical symptoms only (29).

The World Health Organization (WHO) defined vaccination as a “simple, safe, and effective way of protecting you against harmful diseases before you come into contact with them (31).” However, people’s perception of vaccination safety increased compared to before, and that leads to a case of vaccination hesitancy among people. This makes it one of the most controversial subjects among the public. Moreover, the cultural beliefs and the political and religious orientations that they include also affect people’s tendency to take these vaccines (32).

An example of religious beliefs that affect vaccination perception is people who believe that the body is pure and that it should not be contaminated by injecting external substances. There are also those who believe that illness is a divine destiny that should not be interfered with, whether by taking vaccines, taking medications, or through medical procedures (32). In addition, politics can also sometimes affect how people accept vaccines, as there are some countries that give their people complete freedom in their decision-making to take it or not, but this system may not always be in line with ideal public health decisions (32).

Several health behavior theories provide useful frameworks for understanding how cultural beliefs influence vaccination decisions. The Health Belief Model (HBM) posits that an individual’s likelihood of adopting a preventive health behavior is determined by their perceived susceptibility to the disease, perceived severity, perceived benefits and barriers of the action, cues to action, and self-efficacy (33). In the context of HPV vaccination, cultural misconceptions may reduce perceived susceptibility (e.g., the belief that HPV only affects sexually active women), amplify perceived barriers (e.g., concerns about fertility or religious permissibility), and diminish cues to action (e.g., lack of provider recommendations in conservative settings). Similarly, the Theory of Planned Behavior (TPB) suggests that vaccination intention is shaped by attitudes toward the behavior, subjective norms (including family and community influences), and perceived behavioral control (34). Cultural and religious norms, as discussed in the following sections, exert a powerful influence on each of these constructs.

#### Religious and social norms

Religious values can also impact vaccination uptake rates (29). For example, Saudi Arabia’s official religion is Islam. Although the influence of religion on daily life in Saudi Arabia and other Islamic countries has evolved considerably, it continues to shape healthcare-related attitudes, particularly regarding vaccination decisions. In the context of HPV vaccination, both religious and social norms significantly influence public opinion. A recent scoping review of HPV vaccine acceptance in Islamic countries found that some individuals believe vaccines lead to infertility and sexual promiscuity, defy religious norms, may contain haram ingredients, and represent abandonment of righteous principles (35). Vaccine hesitancy in these settings often stems from doubts regarding safety, necessity, and compatibility with religious beliefs.

The religion of Islam is known to highly regard chastity, thus molding the Muslim society into one that values it as well. There are a lot of barriers to HPV vaccine uptake that have arisen from this. Such as, a common belief that is persistent in a certain percentage of the Saudi population has distorted the views on the relation of the HPV vaccination uptake and chastity: many parents fear that the administration of the HPV vaccine can be seen as an approval, or even as an endorsement, for premarital sexual activity (36). Furthermore, other misconceptions about the vaccine include its potential side effects on female fertility. Procreation is an encouraged aspect of Muslim life, and people might fear that the uptake of the HPV vaccine can hinder this deed (37). Therefore, parents might exhibit reluctance when referring to HPV vaccination.

It is essential that policymakers gain the trust of the general public, as their opinion can help shape the overall acceptance of HPV vaccination. An important societal factor to consider is related to religious authorities, who still have substantial impact over societal norms, including vaccination decisions, such that some studies found 45% of people willing to take the vaccine if it were endorsed by religious leaders (38). Thus, policymakers should account for religious sensitivities and emphasize their importance when promoting vaccination practices in various contexts.

#### Role of family and community

Society revolves around certain gender dynamics. The current family system in many developing nations is centered on patriarchal guardianship. This apparent decrease in women’s autonomy can instill the need for their approval as well (39). Moreover, there is a noticeable lack of knowledge about the importance of HPV vaccination due to many reasons, which guides male guardians to select decisions that do not align with the best interests of public health. In general, discussion surrounding sexual health is seen as rather controversial, which leads to the present reluctance toward HPV vaccination due to lack of open dialogue and understanding. This could also be due to lower literacy rates, since some studies show that participants with a higher educational background (43.1%) indicated more awareness of HPV compared to those who had acquired education below the high school level (24.5%) (40). A recent qualitative systematic review by Salleh et al. synthesized parental perspectives across multiple countries, finding that cultural norms and values predominantly shaped vaccination decisions, with fathers often serving as ultimate decision-makers in families where patriarchal structures prevailed, creating significant barriers to vaccinating daughters (41).

### Misconceptions about the HPV vaccine

#### General vaccine misconceptions

Although skepticism surrounding immunization has existed since the development of the first vaccine, there has been an uptick in concern regarding the safety and effectiveness of present-day vaccines (42, 43). Kathy Clift and Denise Rizzolo (42) have indicated that “vaccine safety, religious objections, and skepticism of the science” are the main factors contributing to immunization fears. According to their research, several misconceptions exist among the general population about the safety of vaccines, such as that they could lead to the development of autism, neurodevelopmental disorders, chronic disease, multiple sclerosis, and cancer. Additionally, Rajashri Bezbaruah and colleagues (43) further discuss common myths about vaccines circulating the internet. Examples of such myths include that the DtaP vaccine can cause sudden infant death syndrome, vaccines can induce infertility and are made with toxic materials. Moreover, there are several false notions relating to the effectiveness of vaccines as well. These include beliefs such as vaccines cause the disease they are intended to prevent, natural infection is safer and more effective, vaccine immunity is inferior to natural immunity, and influenza vaccines are overall ineffective (43).

#### Specific myths related to HPV vaccine

Several themes appear throughout the literature on misconceptions about the HPV vaccine. As seen with other vaccines, the HPV vaccine’s safety is often questioned, with many believing that it could lead to infection, cervical cancer, infertility, and sterilization (7, 35, 44–46). There is also speculation on the vaccine’s side effects and overall effectiveness, with some assuming it is in its experimental stages (44, 46, 47). One of the most notable misconceptions about the HPV vaccine is that it is only intended for females, married women, women who are at high risk of cervical cancer, or sexually active women (46, 48, 49). Another common belief among parents, migrant, and isolated communities is that the vaccine promotes promiscuity and is a secretive form of birth control (44). In more religious communities, the HPV vaccine is thought to contain haram, (Islamically prohibited) or unnatural substances (35). A recurrent issue that has become apparent in the literature is the lack of knowledge on the significance of the HPV vaccine in boys (44). Many parents and adolescent males are unaware that the HPV vaccine is recommended and beneficial for boys and assume that the HPV and HPV vaccine is unrelated to males (47, 49). A study at a Saudi University found that 47.1% of male students believed they did not need the vaccination (38).

#### Media influence and misinformation

The spread of misinformation on vaccines was a prominent issue even before social media was established. A prominent example is the now-retracted 1998 study published in The Lancet by Andrew Wakefield, which falsely linked the MMR vaccine to autism (43). Despite its retraction in 2010 and Wakefield’s subsequent loss of medical licensure, the lasting damage to public vaccine confidence has been well documented. Parents hesitated to vaccinate their children, resulting in a measles outbreak. With the rise of social media and the availability of information, individuals can form and share their own views on serious topics, whether accurate or not. Therefore, it has given a platform for “anti-vaxxer” organizations and celebrities to discuss their views on vaccines, often spreading misinformation, governmental and pharmaceutical conspiracies, and alleged negative experiences with vaccines, further persuading individuals to reject immunization (43, 50).

In a study analyzing the impact of social media on vaccine uptake, it was found that negative sentiments on the HPV vaccine resulted in “greater refusal” and, “lower HPV vaccination coverage” (50). However, social media allows for the exchange of ideas on more sensitive topics, and individuals feel more comfortable searching and talking online about the HPV vaccine. Even though positive and educational information on the HPV vaccine is widely discussed in mainstream media and reaches a wider audience, negative content is more likely to be shared on social media. In more traditional countries, such as the United Arab Emirates, media coverage of the HPV vaccine is limited (51). It focuses on informing citizens of the vaccine’s availability and effectiveness, yet shy away from dispelling taboo topics plaguing the HPV vaccine, such as a common belief that it promotes promiscuity.

#### Barriers to HPV vaccination

The transmissibility of the HPV infection is one of the reasons that it has a significant impact on the health and wellbeing of a person. The administration of vaccines has proven to be highly effective, in limiting and preventing HPV-related diseases. Despite the availability and success of these vaccines in protecting against HPV-related diseases, the global vaccination rates are still not as high as they should be (52). Various obstacles spanning issues related to economic disparity, cultural and religious differences, varying health literacy levels, and emotional struggles pose challenges to the widespread acceptance of HPV vaccination efforts among individuals. This part of the analysis aims to dive into these barriers and shed light on the hurdles faced by people in getting access to the vaccine.

### Structural and socioeconomic barriers

Structural barriers are limitations on policies and infrastructure within the current healthcare system that hinder access to the vaccine. Socioeconomic barriers are the financial and social circumstances that influence the person’s ability to receive vaccination.

Issues of preventing HPV vaccination often result from how healthcare systems are organized, how they operate, and the policies and accessibility that govern them in different regions around the world. In low and middle-income countries [LMICs] access to vaccines is limited due to underdeveloped healthcare infrastructure (52). In countries like Saudi Arabia, despite the government’s efforts to improve HPV vaccination rates, the lack of cancer registries and organized systems for tracking HPV vaccination progress make their efforts insufficient in terms of monitoring and enhancing vaccine adoption (53). The lack of organization is not only limited to Saudi Arabia. In fact, it highlights that many countries do not have the proper logistical infrastructure regarding storage, distribution, and administration of the vaccine in both rural and urban areas (54). For example, vaccines that are required to be kept in a cold environment may be harder to transport to rural or underdeveloped areas due to a lack of mobility devices and resources (52).

Infrastructure considerations aside; healthcare policies and the implementation of immunization programs play a role in determining HPV vaccine coverage rates. In some countries where the vaccine is not included in the national immunization program, individuals and families are burdened with financial costs as they have to pay for the vaccines themselves (55). Although most insurance plans in the United States include coverage for HPV vaccines; uninsured individuals encounter difficulties in accessing vaccination services (56). The cost of vaccination may pose a barrier for individuals from disadvantaged socioeconomic backgrounds. This issue is compounded by the perception that HPV vaccination is optional, unlike mandatory immunizations required for school enrollment (46).

Immigrant families living in countries, like Australia and the United States often face challenges when trying to get their children vaccinated against HPV. These neighborhoods or towns ‌face challenges in getting vaccinated due to financial constraints‌ (52). Limited access to medical services‌ ‌due to lack of awareness‌ makes it more difficult to improve vaccination rates.

Boys being left out of immunization programs is an issue that needs addressing urgently because HPV can lead to different cancers, in both genders; yet many countries prioritize vaccinating only girls which has led to lower vaccination rates, among boys and men (49). This gender gap often originates from beliefs that HPV only affects women and is linked mainly to cervical cancer. Studies show that men can also be at risk of developing cancers linked to HPV like anal cancers as well as oropharyngeal cancers (46). It’s important to expand vaccination efforts to include both genders to reduce the effects of HPV.

There are differences in healthcare accessibility between cities and countryside areas. Residents of rural areas frequently face geographic barriers, including long travel distances to healthcare facilities and an insufficient number of healthcare providers. This difference is especially noticeable, in countries where rural healthcare facilities are affected by a lack of funds and a shortage of staff members (53). Without targeted efforts to improve healthcare access, in underserved areas vaccination rates, in these communities may not reach their potential.

### Cultural and psychosocial barriers

As discussed in the preceding section on cultural beliefs and vaccination perception, religious convictions constitute a significant determinant of vaccine acceptance. Building on that foundation, this section examines how these beliefs interact with psychosocial factors to create compounded barriers.

Perceptions of HPV vaccination are substantially influenced by cultural beliefs, societal expectations, and psychosocial factors. A frequent concern is the notion that administering the HPV vaccine to children, specifically girls, could potentially lead to increased sexual activity among them as observed in conservatives societies such as Saudi Arabia and various communities in Western nations, who are hesitant to talk about or provide the HPV vaccine due to worries about it promoting premarital sexual behavior (54). This belief stems from the blending of values and religious beliefs within these communities.

Beyond religiously motivated concerns, evidence indicates that parents in secular societies also express apprehension about administering the HPV vaccine to young children (55). The worry is mainly rooted in the notion that there is a negative link between sexual health and the vaccine. The challenges people often face come from a lack of understanding about the safety and when it is best to administer the vaccine. The vaccine is most effective when taken before becoming sexually active.

Sociopsychological gender perceptions further aggravate barriers in HPV and HPV vaccine uptake. Many parents and individuals still view HPV as a women-centered issue due to its association with cervical cancer. This results in decreased emphasis on young boys being vaccinated, neglecting to address HPV’s link to various cancers affecting men, such as penile, anal, and oropharyngeal cancer (54). In a recent study, it was found that many parents are not well informed about the benefits of giving HPV vaccines to boys, which may explain the low vaccination rates among males (49). This issue is further perpetuated and reinforced by health initiatives that mainly target girls and women, making it appear that the HPV vaccine is not of concern for men.

The willingness of individuals to receive vaccines is greatly impacted by their level of trust in healthcare providers and healthcare facilities. Trust-related issues are especially prominent in minority groups in the United States because of ongoing healthcare access disparities and instances of exploitation that have fueled skepticism toward healthcare establishments. Distrust is often compounded by negative encounters in the healthcare system, such as incidents of discrimination, poor communication, or lack of understanding from healthcare providers (56). Recent research demonstrated how mistrust toward healthcare professionals and pharmaceutical companies hinders the acceptance of HPV vaccination among minorities (57).

Religious beliefs play a major role in shaping opinions about healthcare measures, such as vaccinations. Many religious groups have concerns that the HPV vaccine might promote sexual activity among youth. This is specifically common in conservative and religiously devout communities where discussions about sexual health are often avoided due to social stigma and religious taboos. In Saudi Arabia, for instance, cultural and religious customs pose as obstacles to vaccinating girls due to parents worrying it could wrongly signal approval of promiscuity. It was further noted in a study that the Ugandan community is opposed to vaccinating their daughters, stemming from the misconception that immunization might encourage early sexual behavior (54). The stigma surrounding sexual health issues can impede the acceptance of vaccines, albeit the vaccine being primarily promoted as a means of preventing cancer.

By some in religious groups and communities, it is believed that vaccines go against divine will or disrupt the natural order, which could hinder efforts in HPV vaccination. This belief is not just found in religious settings but also in broader societal circles that are wary that medical interventions are seen as meddling with divine plans. Additionally, in Muslim-majority areas, concerns have arisen about whether the vaccine aligns with religious scriptures and teachings, although religious scholars have agreed that it is permissible. The challenges posed by religion highlight the importance of health education programs that are sensitive to religious beliefs and address concerns related to religion while emphasizing the health benefits of the HPV vaccine in preventing cancer.

### The impact of health literacy

Understanding health information is a crucial aspect of making health decisions. Lack of knowledge or deficits in understanding HPV and the HPV vaccine’s role in healthcare may lead to confusion or prevent individuals from making well-informed decisions concerning their health. In a study, it was found that many young individuals are disoriented by the internet when they have tried to inform themselves about (58). This confusion is a result of the abundant information on the internet, which includes varying information on how HPV spreads and the adverse effects of getting vaccinated. Some people mistakenly believe that HPV only affects women and that the vaccine is necessary for those who are sexually active. This misunderstanding contributes significantly to the reluctance and rejection of vaccines among the population.

In communities with immigrants and minority groups, language barriers make it challenging for people to understand health information. Most of the health education material is in English, making it difficult for non-English speakers to find accurate details about HPV and its vaccine (52). In a study from 2020, it was highlighted that immigrant parents living in non-native countries often lack knowledge about HPV, its vaccine, and the risks associated with not vaccinating their children (52). The lack of understanding is exacerbated by healthcare providers failing to effectively communicate the importance of vaccination in culturally sensitive ways (49).

People who are knowledgeable about health often show interest in getting preventive medical care like vaccines, according to studies (53). Targeted education about the HPV vaccine is essential in enhancing acceptance rates in marginalized groups and increasing vaccination coverage. Health awareness efforts disseminated by credible sources, such as healthcare providers or community leaders, have demonstrated efficacy in enhancing vaccine uptake in regions with historically low vaccination rates.

Health literacy is pivotal when dealing with the great quantity of misinformation about HPV and its vaccine that circulates online. With the increased reliance people have placed on the internet for health information, where both accurate and deceptive content can be found, it can be difficult to navigate. Health educators undermine the necessity of prioritizing literacy to enable individuals to accurately assess health information (58). In the absence of accurate guidance, individuals may find it challenging to differentiate between accurate information and deceptive narratives, perhaps exacerbating vaccine mistrust.

### Strategies to overcome cultural barriers

**Figure 2:**
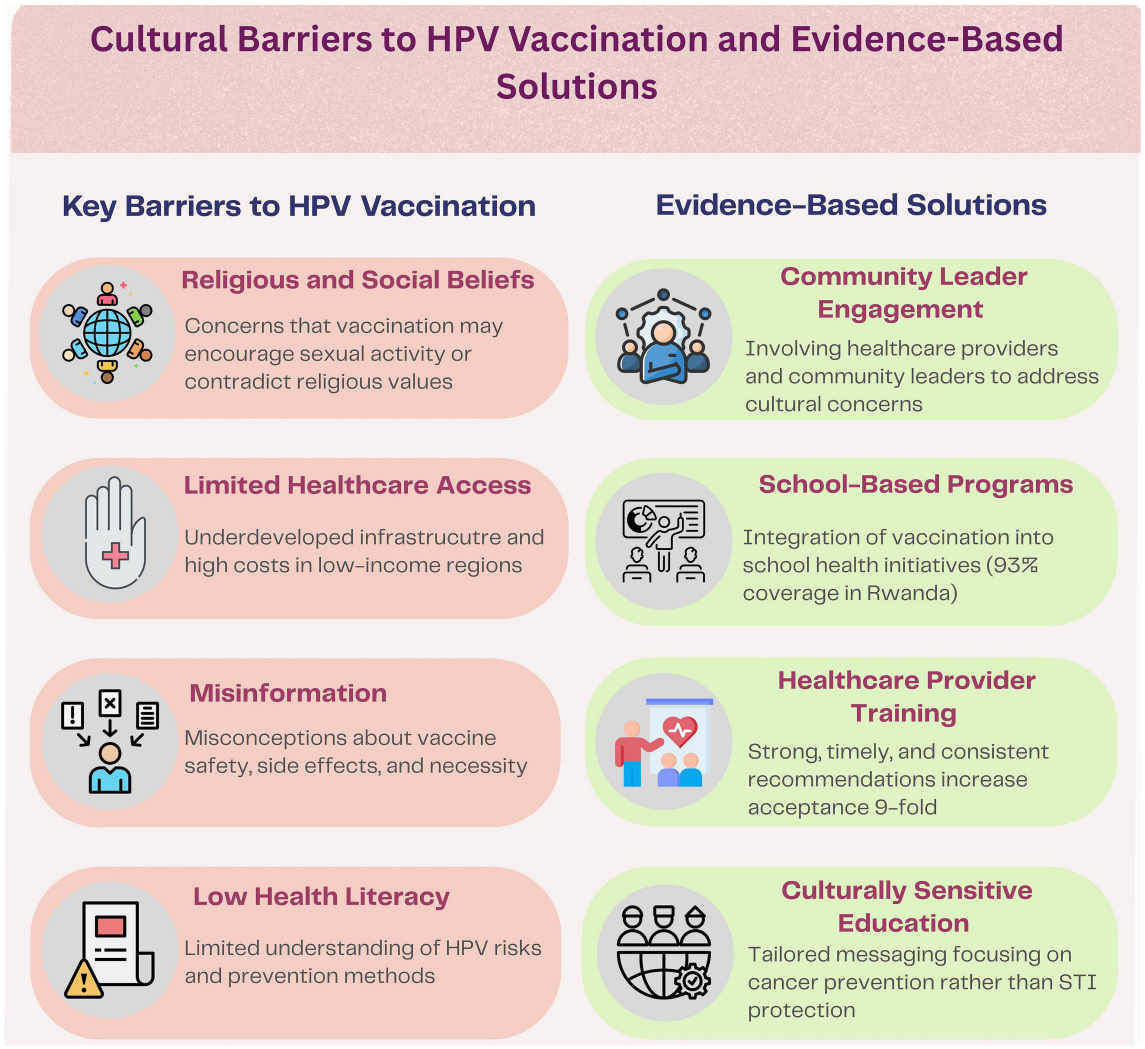
Cultural barriers to HPV vaccination and evidence-based solutions. This figure outlines key cultural and structural barriers impacting HPV vaccination uptake, including religious and social beliefs, limited access to healthcare, misinformation, and low health literacy. Corresponding evidence-based strategies are presented, such as community leader engagement, school-based vaccination programs, targeted healthcare provider training, and culturally sensitive education approaches focusing on cancer prevention.

Figure 2 provides a comprehensive overview of the primary cultural barriers impeding HPV vaccination uptake and corresponding evidence-based interventions that have demonstrated effectiveness in overcoming these challenges.

#### Strategies to improve vaccination rates

Education and awareness initiatives confront false information, debunk myths, and highlight the advantages of HPV vaccination. Targeted campaigns should address common concerns, highlight the long-term benefits of vaccination in avoiding cervical cancer, and provide accurate information about the safety and effectiveness of the vaccines. These campaigns’ reach can be increased by utilizing a variety of platforms, including social media, community workshops, and interactions with healthcare providers, guaranteeing that varied audiences receive correct information (59). Introducing HPV and its vaccine during curriculum visits in schools, improving access to vaccination, and including HPV vaccination in routine vaccination schedules provided by government and private hospitals are a few of the strategies that can foster favorable views toward HPV vaccination and increase its uptake.

Other techniques, such as making the vaccine free and combining HPV vaccination with routine vaccines that are provided in government hospitals and private entities, can improve access to vaccines. Vaccinating schoolchildren as part of governmental vaccination programs decreases HPV burden and has been shown to be effective in multiple countries, including the UAE and Bahrain (3).

Addressing cultural and societal barriers such as misinformation, which has been found to discourage vaccinations, is a powerful tool that aids in reducing HPV burden. Involving local community leaders in awareness campaigns in which they can address misinformation is helpful in building trust and credibility within a community. Primary health care workers can also utilize parents’ visits, where they can thoroughly discuss the HPV vaccine and its importance. Simultaneously, debunking any myths parents may have heard to improve their education and willingness to vaccinate their kids (60).

Success stories from across the world show how effective these strategies are in the prevention of HPV infection and increasing HPV vaccination rates, especially in regions with low uptake. Rwanda’s national HPV vaccination program achieved 93% coverage among sixth-grade girls completing all three doses, representing one of the most successful programs in sub-Saharan Africa (61). The same goes for Uzbekistan, with 94% coverage among 12–14-year-old girls taking the first dose. Non-profit groups and non-governmental organizations also help in providing access to vaccines, vaccinating, and educating individuals in low-income countries such as Vietnam, Uganda, and Peru. Education and communication strategies should not only include high-risk individuals; they need to include the community as a whole, starting from healthcare workers to all parts of the community.

It is critical to align HPV vaccination strategies to cultural values to persuade conservative communities that face cultural barriers to vaccination. Improving vaccination rates by aligning public health initiatives with cultural values involves understanding the community’s beliefs, practices, and norms, then integrating that into health promotion strategies. Engaging community and religious leaders in health promotion campaigns is key to building trust within the community and provides a more targeted campaign promoting vaccination. Designing educational messages in a cultural perspective showed that it can boost vaccination uptake and outlook (60, 61). Highlighting how vaccination can ensure a safer future for mothers and their offspring can significantly improve vaccination outcomes, as it appeals to maternal instincts to take care of their children and protect them from future harm. Addressing myths revolving around HPV and its vaccination using social media is crucial in reaching a larger audience and debunking false notions.

#### Impact of health communication strategies

As misinformation and myths regarding HPV and its vaccine spread among society, it is important to begin educating communities and rebuking myths that have been the major hurdle to immunization and reducing the burden of HPV. Countries introducing the vaccine and mandating new policies should additionally invest in effective communication strategies to increase the uptake of the vaccine. Effective communication methods such as HPV vaccination education and facilitation in vaccine decision-making have been shown to be the most effective in more conservative communities (62). Effective communication strategies can enhance vaccination uptake, address sources of hesitancy, and strengthen routine immunization services (63). Communication helps in building positive attitudes toward HPV and the HPV vaccine, which makes the community more accepting of the idea of vaccination.

Key strategies to improve vaccination rates include community collaboration in the planning and implementation of the vaccine. Collaborative communication can help address any misinformation and enhance prevention education. This idea can be adapted based on the community’s level of literacy and cultural beliefs and adjusted to address specific gender considerations. These strategies help target all members of society, from health care providers to policymakers to schoolchildren.

#### The role of healthcare providers in influencing HPV vaccination decisions

Strong (emphasizing the need for vaccination), timely (recommending age-appropriate and same-day vaccination), and consistent (recommended routinely for all eligible patients) are characteristics of a high-quality recommendation by healthcare providers (64–66). Studies have shown that parents who received recommendations from healthcare providers are 10 times more likely to get their children vaccinated (30, 65, 67, 68). The recommendation’s quality is also important. According to one study, parents who received a strong, timely, and consistent recommendation for the HPV vaccine had a greater than nine-fold chance of accepting the vaccine for their child compared to parents who did not receive one. According to Melissa Gilkey and colleagues (64), parents who received a recommendation of low quality, like misleading information or unconvincing facts, were only four times as likely to consent to their child receiving the vaccine.

#### Current policies in the Middle East and North Africa region

Bahrain recently announced that HPV vaccination is now part of the routine vaccine schedule for females aged 9 and older, as well as for women aged 25–45, to reduce the burden of HPV as an infectious disease and a leading cause of cancer. Since October 2023, the country has also targeted boys and girls aged 12–13, as part of a school vaccination campaign. Other countries in the region have similar initiatives. Iran offers the HPV2 and HPV9 vaccines to high-risk groups aged over 25 years old, while Kuwait provides the HPV9 vaccine to males and females aged 9–45. Libya vaccinates 12-year-old females with HPV4 in a series of three doses. Qatar offers HPV9 to males and females aged 11–26, with two doses for younger adolescents (9–14) and three doses for those 15 and older. In Saudi Arabia, high-risk groups receive HPV4 at ages 11 and 12, and in the UAE, schoolchildren aged 13 are given HPV9 (69). However, since all of these mandates are newly issued, it is unknown how effective they are. In the next few years, we will be able to determine whether these mandates have been effective in preventing HPV and increasing the uptake of the vaccine in these conservative regions.

### Case studies and comparative analysis

#### Regional case studies

The comparative analysis of HPV vaccination uptake rates in developed versus developing countries gives us information about the various factors that influence these rates, such as education, socioeconomic status, and awareness. When we compare the Western countries with more conservative regions, the difference in HPV vaccination uptake rates highlights the complex interplay of cultural, social, and economic factors that significantly influence public health decisions.

In the United States, geographic and demographic factors play an important role in determining the differences in initiation and completion rates of HPV vaccination. According to a study conducted in the US, women living in the South and West were less likely to initiate and complete the three-dose HPV vaccine series compared to those in the Northeast (70). This difference in the rates of uptake and completion of doses in these regions is further influenced by socioeconomic factors, such as those individuals that come from lower-income backgrounds often facing barriers such as limited access to healthcare, misinformation, and cultural stigmas that hinder their process and ability to receive the vaccine.

Although there has been an overall increase in HPV vaccination rates among young adult women all over the US, young adult men, however, are still lagging behind with low initiation and completion rates. This highlights the necessity for focused interventions that tackle concerns and barriers specific to different genders. In many developing countries, the circumstances are frequently more complicated. A qualitative study conducted in Iran revealed that many women did not know the importance of screening for prevention of cervical cancer among women and had limited knowledge and low awareness about cervical cancer. Many participants were influenced by a lot of misconceptions that negatively influenced their uptake of preventive measures, and they also expressed a low sense of vulnerability to cervical cancer (71). This lack of awareness is not exclusive to Iran; it reflects a wider pattern observed in many developing countries where education systems do not prioritize sexual health education.

Research examining HPV vaccination across Gulf Cooperation Council (GCC) countries has revealed notable variation in uptake rates. A 2024 comparative cross-sectional survey examining HPV vaccination across all six GCC countries (Bahrain, Kuwait, Oman, Qatar, Saudi Arabia, and UAE) among 831 young adults aged 18–39 revealed notable variation in vaccination rates (72). The UAE demonstrated the highest vaccination rate at 18.9%, followed by Qatar at 5.8% and Saudi Arabia at 4.6% (*p* < 0.001). Health insurance status emerged as a significant determinant: insured individuals had higher vaccination rates than uninsured counterparts (11% vs. 5.4%, *p* = 0.006). The primary barriers identified were lack of knowledge (53.6%) and absence of medical recommendations (13.2%), highlighting the critical role of healthcare provider engagement and public education (69, 72, 73). As we discussed earlier, cultural factors also play an important role in determining HPV vaccination rates. In Singapore, a study revealed that 76% of respondents believed that cultural practices influenced health decisions, with religion, nationality, and ethnicity being primary determinants (47). These cultural dynamics which are further influenced by the concerns of stigma that exist in the society or the misconceptions about sexual health can lead to resistance to vaccination.

In increasing awareness and reducing misconceptions, myths related to HPV vaccination education plays a very crucial role. A study carried out at King Saud Medical City in Saudi Arabia revealed a significant link between individuals’ education levels and their understanding of HPV and cervical cancer screening methods. The participants who had lower levels of education showed lack of knowledge about PAP tests and the HPV vaccine, calling our attention to the importance of educational programs to improve vaccination rates (48). Moreover, a recent study showed that up to 45% of female healthcare workers in Saudi Arabia were willing to take the HPV vaccine, and one-fifth had already received at least one dose. This increase is largely due to enhanced awareness through health campaigns and community outreach initiatives (74). This finding emphasizes the importance of educational efforts and awareness in increasing vaccination rates.

A recent study conducted by Alghalyini et al. (73) examined HPV awareness, knowledge, and vaccine acceptability among 442 college students in Saudi Arabia. Their findings revealed that while 54.1% of participants were aware of HPV and 66.5% had heard of cervical cancer, only 17% correctly identified the high-risk genotypes HPV16 and HPV18 as primary causes of cervical cancer. Knowledge disparities were evident between genders, with females demonstrating significantly higher knowledge levels than males. Notably, only 36.2% of participants reported awareness of the HPV vaccine, with a mere 10% having received vaccination. The researchers identified lack of education and awareness (80.1%) as the primary barrier to vaccination uptake, highlighting the urgent need for targeted educational interventions tailored to cultural sensitivities, especially to bridge the knowledge gaps between genders and education levels.

Addressing the gaps in HPV vaccination uptake requires a comprehensive strategy that considers the complex relationships between education, socioeconomic factors, and cultural influences. Countries can enhance HPV vaccination rates and therefore, reduce the incidence of HPV-related diseases by taking health initiatives to target these barriers and utilizing community resources for education and outreach. To improve public health on a global scale, it is also essential for policymakers and public health officials to prioritize education and readily available healthcare services to empower decision-making among individuals.

#### Comparative analysis

As noted in the barriers section above, Taghizadeh Asl et al. (71) demonstrated that Iranian women exhibited inadequate knowledge of cervical cancer screening, compounded by misconceptions regarding hereditary risk and confusion between STIs and cervical cancer. Societal taboos surrounding sexual health discourse, combined with limited healthcare provider engagement in screening promotion, constituted significant barriers. The authors emphasized that improving health communication and dismantling socio-cultural barriers within the Iranian healthcare system are prerequisites for enhancing screening behavior (71).

A Saudi secondary school girls’ study, focusing on educational intervention to enhance knowledge of the HPV vaccination and myth debunking, showed that the participants’ intention to get vaccinated was immensely enhanced, as had their level of understanding about HPV and its connection with cervical cancer. Most of the girls had previously been given scant or incorrect information; examples include the belief that the vaccine is only for women and an explanation of how the vaccine will prevent cervical cancer. These fallacies were dispelled through the instructional lectures, thus increasing the rate of vaccination acceptance. Cultural and religious sensitivities were noted to remain a barrier to acceptance in the study. The intervention thus performed quite well in raising awareness, but the deep-seated social attitude changes toward sexual health remain tough in Saudi Arabia (75).

Research conducted in 2023 has provided a comprehensive review of health education programs in India for young people’s acceptance, awareness, and uptake of the HPV vaccination (76). Analysis revealed that while health education was effective in improving knowledge and awareness, several barriers to vaccination uptake were identified. The prevailing barriers included misinformation, stigma around sexual health, and costs associated with the vaccine. There was indeed an improvement in the level of understanding of the subjects and a tendency toward vaccination after the educational activities; however, practical efficacy was limited by conventional social attitudes and economic concerns. The study lists the need for focused information programs which would consider such obstacles in rural and urban settings and accessible vaccine availability (76).

In conclusion, narrowing the gaps in vaccination against HPV requires an engaging plan that considers the interrelated factors of education, socioeconomic status, and cultural background. Vaccination will not only reduce the burden of diseases associated with human papillomavirus infection but also contribute to health measures by breaking these barriers with the assistance of community resources in education and outreach. Real behavior change, be it in screening participation or vaccination uptake, requires a multi-dimensional approach in terms of available health care choices, culturally sensitive outreach, and vigorous engagement on the part of healthcare providers and governments. In order to make personal responsibility an informed choice possible and enhance the global outcomes of public health, education and free health services must be brought to the fore by policymakers and public health professionals.

#### Strengths and limitations

This narrative review offers several strengths. It provides a multi-regional synthesis of cultural barriers to HPV vaccination, drawing on evidence from diverse geographic and sociocultural contexts including the Middle East, sub-Saharan Africa, South and Southeast Asia, and Western nations. This breadth facilitates cross-cultural comparisons that may inform context-specific interventions. The review also adopts an interdisciplinary perspective, incorporating sociological, religious, psychological, and public health dimensions of vaccine hesitancy. Lastly, the identification of evidence-based strategies linked to specific cultural barriers provides actionable guidance for policymakers and healthcare practitioners.

Several limitations should be acknowledged. As a narrative review, this study did not employ a systematic search protocol with formal quality assessment of included studies, which limits the reproducibility and comprehensiveness of the evidence synthesis. Publication bias may have influenced the literature identified, as studies reporting significant cultural barriers are more likely to be published than those finding no association. The restriction to English-language publications may have excluded relevant research from non-English-speaking regions where cultural barriers are particularly pronounced. Additionally, the heterogeneity of study designs, populations, and outcome measures across included studies precluded quantitative synthesis or meta-analysis. The subjective nature of thematic synthesis in narrative reviews introduces the possibility of interpretive bias. Finally, the rapidly evolving policy landscape in the MENA region means that some of the policy information presented may have changed since the literature search was conducted.

## Conclusion

This narrative review demonstrates that cultural beliefs and misconceptions represent formidable yet modifiable barriers to HPV vaccination uptake worldwide. The interplay of religious values emphasizing chastity, patriarchal family structures limiting women’s healthcare autonomy, persistent myths regarding vaccine safety and fertility, and gendered perceptions of HPV as a female-only concern collectively undermine vaccination efforts across diverse settings. These challenges are further compounded by structural barriers including inadequate healthcare infrastructure, prohibitive costs, and insufficient health literacy, particularly in low- and middle-income countries. Evidence from regional case studies demonstrates that culturally tailored interventions can substantially improve vaccination outcomes. Rwanda’s school-based program, healthcare provider training initiatives, religious leader engagement, and targeted educational campaigns have each shown measurable success in specific contexts. The consistent finding that strong, timely, and consistent provider recommendations increase vaccine acceptance up to nine-fold underscores the critical role of healthcare professionals in this effort.

Future research should prioritize longitudinal studies examining how cultural attitudes toward HPV vaccination evolve over time, intervention trials comparing the effectiveness of different culturally adapted strategies, and mixed-methods studies that capture the lived experiences of under-vaccinated populations. Greater attention to male-focused research, rural and underserved populations, and the impact of digital misinformation is also warranted. Achieving the WHO’s target of 90% HPV vaccination coverage by 2030 will require sustained, context-sensitive, and culturally informed public health approaches that leverage community trust and address local misconceptions.
